# Editorial: Current challenges and future perspectives in neuro-oncological imaging

**DOI:** 10.3389/fradi.2025.1731279

**Published:** 2025-11-21

**Authors:** Emma Gangemi, Paola Feraco, Carlo Augusto Mallio

**Affiliations:** 1 Radiology and Diagnostic Imaging, IRCCS National Cancer Institute Regina Elena, Rome, Italy; 2 Department of Human Neurosciences, Sapienza University of Rome, Rome, Italy; 3 Centre for Medical Sciences (CISMed), University of Trento, Trento, Italy; 4 Fondazione Policlinico Universitario Campus Bio-Medico, Rome, Italy; 5 Research Unit of Diagnostic Imaging, Department of Medicine and Surgery, Università Campus Bio-Medico di Roma, Rome, Italy

**Keywords:** brain tumors, neuro-oncological imaging, precision medicine, quantitative imaging, artificial intelligence

Over 100 distinct types of primary CNS tumors contribute to a variety of histopathological and molecular profiles, each with unique clinical presentations, treatment strategies, and prognostic implications. Recent advancements in molecular diagnostics, along with traditional histology and immunohistochemistry, have enhanced our understanding of the histogenetic classification of these tumors, as reflected in the latest WHO classification (5th edition) of brain tumors (1). Despite therapeutic advancements, the prognosis for patients with brain tumors, particularly those with high-grade neoplasms, remains poor. The heterogeneity and complexity of these tumors underscore the need for personalized, targeted treatment approaches. Quantitative MRI (qMRI) and Artificial Intelligence (AI) are key tools in modern neuro-oncology, providing advanced diagnostic and prognostic capabilities (2–4). qMRI quantifies diffusion, perfusion, and metabolism, while AI—using Deep Learning and Radiomics—automates tumor analysis and integrates imaging with genetic and clinical data. Together, they improve accuracy, reproducibility, and treatment planning, driving progress in precision medicine for brain tumors (5–7). Automated volumetric assessment reduces interobserver variability and predicts survival more reliably than manual measurements (8, 9).

The goal of this Research Topic was to review recent advancements in neuro-oncological imaging modalities and their impact on managing brain tumors. By focusing on innovations in diagnosis, cancer staging, prognostication, pre-treatment assessment, and treatment monitoring, we aimed to highlight the enhanced accuracy and effectiveness these tools bring to neuro-oncology, optimizing personalized healthcare strategies.

Ghankot et al. examined how MRI voxel resolution affects the precision of tumor volume measurements in Neurofibromatosis type 2 related Schwannomatosis (NF2-SWN). A total of 17 tumors (seven bilateral and three unilateral) vestibular schwannomas (VS) were segmented using both manual and AI-based methods, and the effect of voxel size on segmentation precision was quantified through volume measurements. Across the dataset, the authors observed a clear inverse relationship between voxel size and segmentation accuracy. Smaller voxel sizes (e.g., 0.5 × 0.5 × 0.8 mm) significantly improved the accuracy and consistency of both manual and AI-based tumor segmentation, while larger voxels reduced precision. AI segmentation was more robust and showed reduced variability than manual methods, especially at lower resolutions. The study concludes that high-resolution MRI and AI-driven segmentation are essential for reliable tumor monitoring and treatment planning in patients with NF2-related vestibular schwannomas.

The review by Cataldi et al. summarizes how intra-tumoral susceptibility signals (ITSS) on susceptibility weighted imaging (SWI) sequences, reflecting microhemorrhages, calcifications, necrosis, and vascular proliferation in gliomas, correlate with tumor grade and molecular markers (IDH, MGMT, ATRX, Ki-67). Moreover the paper underlines the usefulness of emerging quantitative and AI-based methods for the ITSS assessment. Quantitative Susceptibility Mapping (QSM) is emerging as a promising tool to overcome the limitations of semi-quantitative methods, by providing quantitative measures of magnetic susceptibility, eliminating operator dependency and allowing to differentiate between distinct sources of ITSS, such as hemorrhage, calcification, and other susceptibility effects. Radiomics and artificial intelligence are enhancing the evaluation of ITSS, enabling a deeper analysis of ITSS characteristics like different vascular abnormalities or iron deposition, addressing tumor subtypes classification and prediction of aggressiveness or response to treatment with high accuracy.

The need for personalized healthcare strategies in the neuro-oncology field is underscored in the study by Mallio et al., that investigated the relationship between radiation necrosis and lesion location in patients treated with fractionated stereotactic radiotherapy for brain metastases. Among 167 lesions in 41 patients, 25% developed radiation necrosis. Lesions in the deep-periventricular region and those near neural stem cell niches showed a significantly higher risk of necrosis. These findings suggest that specific brain regions and proximity to stem cell niches influence tissue vulnerability to radiation damage, highlighting the need for region-sensitive radiotherapy planning and potential neuroprotective strategies.

The usefulness of several imaging features for the prediction of biological behavior in brain tumors was shown in the paper by Zhang et al. The authors analyzed 69 cases of intraventricular central neurocytomas (CNs) and explored how tumor burden varies with the increase of the Ki-67 proliferation index. CNs with high Ki-67 (>3%) were larger, more solid, and showed restricted diffusion, CT hyperdensity, abnormal vessels, and marked MRI enhancement compared with low Ki-67 tumors. These findings indicate that tumor cellularity and vascularity increase with Ki-67, reflecting more aggressive biological behavior.

The role of advanced MRI techniques and in particular of brain functional connectivity in tracking the effects of surgical treatment on patient cognitive functioning was explored in the study by Ellis et al. The authors examined 21 patients who underwent MRI before and two weeks after brain tumor resection, finding that changes in functional brain connectivity, especially local efficiency, global efficiency, and modularity, were strongly correlated with changes in neuropsychological test scores. These results suggest that functional connectivity metrics may serve as non-invasive biomarkers to monitor and predict cognitive outcomes after brain surgery.

Finally the research topic incorporates two detailed and highly relevant case reports. Zhang et al. report two cases of anaplastic papillary glioneuronal tumor (PGNT), one of which progressed to glioblastoma (WHO Grade IV). The study highlights that advanced MRI biomarkers diffusion restriction, and metabolic changes together with edema, cyst wall enhancement and hemorrhage, can help predict malignant potential in anaplastic PGNT. Early recognition may guide surgical planning and adjuvant therapy. Xu et al. describe the first known human case of a microcystic meningioma (MM) located in the fourth ventricle, in a 54-year-old man. The tumor was completely resected, and histopathology confirmed MM. One year later, imaging revealed a new lesion at the surgical site, which was surgically removed and diagnosed as a giant cell reparative granuloma, a rare reactive lesion likely caused by surgical trauma. This case highlights the importance of accurate imaging-based differential diagnosis for atypical meningioma locations and regular postoperative follow-up to detect reactive or recurrent lesions early.

In summary, the studies featured in this Research Topic collectively emphasize how advanced MRI techniques and quantitative analytics are transforming neuro-oncological imaging. These innovations are oriented towards more precise diagnostics, individualized treatment planning, and ultimately, improved patient outcomes in brain tumor management.
